# Missing data and prediction: the pattern submodel

**DOI:** 10.1093/biostatistics/kxy040

**Published:** 2018-09-06

**Authors:** Sarah Fletcher Mercaldo, Jeffrey D Blume

**Affiliations:** 1 Department of Radiology, Institute for Technology Assessment, Massachusetts General Hospital, 101 Merrimac St., Suite 1010 Boston, MA, USA; 2 Department of Biostatistics,Vanderbilt University, 2525West End, Suite 1100, Nashville, TN, USA

**Keywords:** Missing data, Missing-indicator method, Pattern Mixture Models, Prediction models

## Abstract

Missing data are a common problem for both the construction and implementation of a prediction algorithm. Pattern submodels (PS)—a set of submodels for every missing data pattern that are fit using only data from that pattern—are a computationally efficient remedy for handling missing data at both stages. Here, we show that PS (i) retain their predictive accuracy even when the missing data mechanism is not missing at random (MAR) and (ii) yield an algorithm that is the most predictive among all standard missing data strategies. Specifically, we show that the expected loss of a forecasting algorithm is minimized when each pattern-specific loss is minimized. Simulations and a re-analysis of the SUPPORT study confirms that PS generally outperforms zero-imputation, mean-imputation, complete-case analysis, complete-case submodels, and even multiple imputation (MI). The degree of improvement is highly dependent on the missingness mechanism and the effect size of missing predictors. When the data are MAR, MI can yield comparable forecasting performance but generally requires a larger computational cost. We also show that predictions from the PS approach are equivalent to the limiting predictions for a MI procedure that is dependent on missingness indicators (the MIMI model). The focus of this article is on out-of-sample prediction; implications for model inference are only briefly explored.

## 1. Introduction

### 1.1. The problem

Missing data are problematic for both estimation and prediction. The statistical literature has been focused on addressing the impact of missing data on parameter estimation and inference. However, forecasting with incomplete predictor information is also highly problematic. The misspecification of an influential predictor can be very costly in terms of prediction accuracy. Thus, it is important to have a validated and robust approach for handling such instances.

In this article, we focus on what Wood *and others* (2015) call pragmatic model performance: the model’s performance in a future setting where records may have partly missing predictors. For context, we consider the scenario of building clinical prediction models for use in hospital or out-patient settings. But these methods are broadly applicable to any prediction setting. The problem arises when one attempts to apply an established prediction algorithm to a new (out-of-sample) individual who has an incomplete predictor profile (i.e. some predictors are missing). It is not hard to imagine that the manner in which those missing predictors are dealt with is critical for maintaining the predictive accuracy of any forecast.

It is often assumed that “proper” imputation of new individual’s missing predictors will, at the least, maintain the predictive accuracy of any algorithm. Some have even claimed that certain types of imputation can improve the accuracy of forecasts. However, we could find no rigorous justification for these claims and our investigation indicates that this would be the exception rather than the rule. As we describe below, imputation methods can maintain the predictive accuracy of a model when key assumptions about the missing data mechanism are met. The predictive accuracy of our proposed approach, pattern submodels (PS), is at least as accurate as imputation methods and does not depend on assumptions about the missing data mechanism. In many cases, it is also more computationally efficient.

In our view, the impact of missing data on out-of-sample prediction performance is uniformly understated in the statistical and clinical literature. A poor imputation algorithm, e.g. zero-imputation or mean imputation, can drastically reduce a model’s prediction performance in practice. This a highly practical problem with wide applicability.

### 1.2. Current approaches to imputing missing predictors

When applying prediction algorithms, strategies for dealing with missing predictors are often driven by practical constraints. Common strategies include zero imputation and mean imputation, which are trivial to implement, but often lead to poor predictions when the missing predictors are influential. Conditional mean imputation and multiple imputation (MI) are now regularly implemented when fitting a model, but they are rarely used in real time when the model is applied to new individuals that are missing predictors (Janssen *and others*, 2009). The advantages and drawbacks of imputation methods for out-of-sample imputation are listed in Table 1. The obvious issue, not well addressed in the literature, is the extent to which these approaches degrade prediction performance.

**Table 1.** Comparing imputation methods for an out-of-sample individual with missing data

	Out-of-sample Imp.		
	Requires	Pros	Cons
Zero imputation	Nothing	Neglible computation time	Zero may not be an appropriate value
			Probably results in incorrect predictions
Mean imputation	Unconditional means	Neglible computation time	Only works for the average individual
Conditional mean imputation	Conditional mean imputation model for every missing data pattern	Lower computation time	Large bias/variance tradeoff for MNAR
		Can approximate a MI procedure	
CCS	Submodels to be fit	Negligible computation time	Large bias/varaince tradeoff for MNAR
		May be advantageous if data are MAR	
		Fittable for unobserved patterns	
PS	Submodels to be fit	Negligible computation time	May be less efficient if data are MAR
		Works for any missingness mechanism	Patterns with low membership may not fit well
MMI	Original data/conditional distribution	Works for any missingness mechanism	High computational cost
	Computer/imputation engine	Allows for efficient parameter estimation	Not viable in the clinic
MI	Original data/conditional distribution	Established method	High computational cost
	Computer/imputation engine	Works when data are MAR	Not viable,in the clinic
			Large bias/variance tradeoff for MNAR

MI is typically used in the model construction stage. MI draws multiple placeholder values from conditional distributions derived from the observed data (van Buuren, 2012; Janssen *and others*, 2010; Harrell, 2013) and uses those placeholder values to fit the model. The coefficients from each of these fits are combined using Rubin’s rules (Rubin, 2009). When the data are missing at random (MAR), MI can substantially increase the efficiency of inferential procedures by leveraging partial information from incomplete data records. The “best” predictions from a multiply imputed prediction model are the averaged predictions over all imputation sets (Vergouwe *and others*, 2010; Wood *and others*, 2015). The popularity of MI for handling missing data in both the construction and forecasting stages has recently grown (Harrell, 2013; Janssen *and others*, 2009).

However, the applicability of the approach described above is limited when the user only has access to published parameter estimates. Making predictions from a multiply imputed model when predictors are missing is not straightforward, and it generally requires access to the original data in addition to the published parameter estimates. In order for the real time imputations to be reflective of the MI procedure used during model construction, the additional out-of-sample record should be combined with the original data, and the full imputation algorithm should be refit to properly fill in missing predictors.

This nuance is especially important when using predictive mean matching or K-nearest neighbor imputation techniques (as we do here). Of course, this requires access to the original data, the imputation datasets, and substantial on-demand computing power, which is typically impractical in real world settings. Moreover, because of its heavy computational burden, MI is not easily incorporated into web applications. One option is to simply ignore this issue and use the multiply-imputed model along with a one-step imputation procedure rooted in the chain equations or fitted conditional distributions. But this process is not likely to be congenial with the original fitting approach and it did not perform well in our investigations.

### 1.3. Proposed solution

Our proposed solution is to use an approach we call the PS procedure. The basic idea is to fit a pattern mixture model and forecast from whichever pattern-specific model matches the new but incomplete predictor profile. This simple idea turns out to be highly effective and quite flexible. How the PS are fit is important; we suggest using data only from that pattern to avoid having to make assumptions about the missing data mechanisms. Note that the fitting of PS requires no imputation because standard model construction typically ignores predictors that are uniformly missing in an entire group. Details are provided in Section 3.1. Forecasts from PS benefit from the reduction in prediction bias that comes with the pattern-specific approach. The loss in efficiency in parameter estimation that can result often translates to a relatively minor penalty in prediction measures. Moreover, as we show later, the PS approach is optimal in the sense that it minimizes the expected prediction loss in the class of models being fit. Because of these advantages, we anticipate that the PS approach will have broad impact for big data applications where prediction is paramount and MI is often computationally unfeasible, e.g. when using an entire system of electronic medical records (EMR).

### 1.4. A notable relationship

There are some interesting mathematical connections between PS and MI. PS is the limit of a congenial MI procedure where the mean model depends on the missing data indicators. This new MI procedure, which we call multiple imputation with missingness indicators (MIMI)—gives limiting predictions that are equivalent to the PS models. What is nice about the MIMI model is that it makes it clear what elements of the model can be assumed identical across the patterns. However, to forecast from the MIMI model requires imputation and therefore substantial computational commitment.

The MIMI model is also known as the missing indicator model and is known to perform poorly for estimation purposes when simple imputation methods such as zero or mean imputation are employed (Allison, 2001; Groenwold *and others*, 2012). However, a corollary of our approach is that the inclusion of the missing data indicator in the mean model is not the root problem; rather it is how the missing data placeholder is imputed that causes subsequent bias in parameter estimation. When MI is used to fill in missing values in a MIMI model, the parameter estimates will remain unbiased under certain conditions on the missing data mechanism. The lesson is that the utility of the missing data indicators can only be realized through an imputation procedure; an often overlooked point. We will not explore this connection in this article, but the connection is important to note in this context.

### 1.5. Organization

Section 2 defines our notation and provides essential background on key missing data concepts. Section 3 describes our proposed methods, provides a simple example, and draws connections between PS and MI models in some generality. Section 4 describes extensive simulations of PS in comparison to standard approaches. Section 5 describes the performance of PS compared with other imputation strategies applied to the SUPPORT Study, a multi-center two phase study of 9105 patients, from which a day 3 Physiology Score (SPS) was predicted (Knaus *and others*, 1995). Section 6 provides some brief concluding remarks.

## 2. Notation and background

### 2.1. Notation

Let $\boldsymbol{Y} = (Y_1,...,Y_n)$ be the vector of $n$-length observed responses. With our focus on prediction, we assume all responses are observed. This assumption can be relaxed, but it is not pertinent to our discussion here. Predictors (covariates) are denoted by a $(n \times p)$ matrix $\boldsymbol{X} = (\boldsymbol{X_1},...,\boldsymbol{X_p})$ where $\boldsymbol{X_j} = (X_{1j},...,X_{nj})^T$ for $j = 1,...,p$ predictor vectors of length $n$. Let $\boldsymbol{M} = \{M_{ij}\}$ be the $(n \times p)$ matrix of missing data indicators where indicator $M_{ij} =1$ if $X_{ij}$ is missing and $M_{ij} =0$ if $X_{ij}$ is observed for $i = 1,...,n$ individuals and $j = 1,...,p$ parameters. This notation is consistent with Dardanoni *and others* (2015) and White and Carlin (2010), two of our key references, but reversed from the more common notation where observed and missing predictors are assigned the values 1 and 0, respectively. $\boldsymbol{X_M}$ is used to indicate the subset of all available predictors in missing data pattern $\boldsymbol{M}$.

To help differentiate between models, we will use different Greek symbols for their parameters. For example, parameters in the PS will be donated with a $\boldsymbol{\gamma}$. Parameters in a traditional regression model $E[Y|\boldsymbol{X}] = \boldsymbol{X}\boldsymbol{\beta}$ will be donated by $\boldsymbol{\beta}$; these are the parameters that are typically of interest in an estimation setting. Note that the assumption of a common mean model across all missing data patterns (i.e. some version of MAR) is obviously a strong one. Parameters representing the effects of the missingness indicators, $\boldsymbol{M}$, will be denoted by $\boldsymbol{\delta}$; these parameters distinguish the MIMI model (defined in Section 3.4) from a traditional MI model.

### 2.2. Background on pattern mixture and selection models

Our approach has roots in the established literature on pattern mixture models (Little, 1993). The pattern mixture model factorization is: $P(\boldsymbol{Y},\boldsymbol{M}|\boldsymbol{X},\boldsymbol{\gamma},\boldsymbol{\pi}) = P(\boldsymbol{Y}|\boldsymbol{X},\boldsymbol{M},\boldsymbol{\gamma})P(\boldsymbol{M}|\boldsymbol{X},\boldsymbol{\pi})$, where $\boldsymbol{\pi}$ is a parameter vector for the missingness mechanism (Little and Rubin, 2014). The pattern-mixture approach allows for a different response (mean) model in each missing data pattern. Our PS models are the kernels $ P(\boldsymbol{Y}|\boldsymbol{X},\boldsymbol{M},\boldsymbol{\gamma})$ of the pattern-mixture model. By fitting a submodel using only data from that missing data pattern, we avoid the reliance on assumptions about the forms of the missing data mechanism. An alternative formulation is the selection model: $P(\boldsymbol{Y},\boldsymbol{M}|\boldsymbol{X},\boldsymbol{\theta},\boldsymbol{\omega}) = P(\boldsymbol{Y}|\boldsymbol{X},\boldsymbol{\theta},\boldsymbol{\omega})P(\boldsymbol{M}|\boldsymbol{Y},\boldsymbol{X},\boldsymbol{\omega})$ where $\boldsymbol{\theta}$ and $\boldsymbol{\omega}$ are parameter vectors (Little and Rubin, 2014; Little and Wang, 1996). This factorization describes a (single) marginal response model, possibly dependent on elements from of the missing data mechanism. In this article, we will not explicitly consider selection models except to use them to simulate data from certain missing data mechanisms. While the selection model allows for potential dependence of $\boldsymbol{Y}$ and $\boldsymbol{M}$, the pattern-mixture model is traditionally used when the response model changes by missing data pattern.

### 2.3. Missingness mechanisms

To describe missing data processes, we define the following mechanisms: missing completely at random (MCAR; $\boldsymbol{M} \sim c$ for some constant $0<c<1$ ), missing at random (MAR; $\boldsymbol{M} \sim \boldsymbol{X_{obs}}$), missing not at random (MNAR; $\boldsymbol{M} \sim \boldsymbol{X_M}$), MAR where the missingness depends on $Y$ (MARY; $\boldsymbol{M} \sim \boldsymbol{X_{obs}} + Y$), and missing not at random where the missingness depends on $Y$ (MNARY; $\boldsymbol{M} \sim \boldsymbol{X_M} + Y$) (White and Carlin, 2010; Little and Rubin, 2014). The latter two mechanisms can only be simulated in the selection model formulation.

If the missingness mechanism is MCAR, then pattern mixture and selection models are equivalent (Little, 1993). When the data are not MCAR, the parameters of the kernel functions associated with the selection and pattern mixture models have different interpretations and care must be taken when estimating and interpreting them. The selection model describes the marginal relationship of $\boldsymbol{Y}$ on $\boldsymbol{X}$, while the pattern mixture model describes the relationship of $\boldsymbol{Y}$ on $\boldsymbol{X}$ conditional on $\boldsymbol{M}$. Marginal effects from the selection model are generally not identifiable in the context of a pattern mixture model, although some parameterizations can be identified though complete case restrictions that essentially force equality restraints on certain parameters (Little, 1993). Identifiability is obviously a problem when the goal is estimation and data are MNAR. However, when forecasting is the goal, complex re-parameterizations of marginal effects are not a major impediment, even if the mapping is not easily reversed. If a single marginal model is truly of interest, one can always marginalize over the pattern-specific models. Of course, how that model should be interpreted when the data are not MAR is not immediately clear.

### 2.4. Complete case approaches

A complete case analysis simply ignores the records with missing data and estimates a single model from the complete data set. Complete-case models have poor prediction performance when the data are not MCAR (Knol *and others*, 2010; Janssen *and others*, 2009). Moreover, the comple-case model still requires some type of imputation when forecasting from incomplete records.

The complete case analysis should not be confused with complete case submodels (CCS). CCS are similar to our proposed PS approach in that they fit a unique model for every missing data pattern. However, CCS use data from all patterns to estimate the fit in each pattern. This often entails re-using records and discarding observed data. For example, if age were the only missing predictor, two models would be fit: one with age and one without. The PS approach would fit each model submodel separately using the set of records with age and the set of records without age. CCS, on the other hand, uses all the records to estimate the submodel that excludes age; age is simply ignored in the records that have it. As a result, the validity of CCS predictions depends on a very strong MCAR assumption, which is often violated in practice. CCS has been explored by Janssen *and others* (2009) and it is the only other submodel approach that we found that has been systematically examined.

## 3. Methods

### 3.1. Pattern submodels

Let $\{\hat{f}_1,...,\hat{f}_k\}$ where $\hat{f}_m = \hat{f}_m(\boldsymbol{X_M},\boldsymbol{M})$ be the set of pattern-mixture submodels for pattern $m = 1,...,k$ where $k \leq 2^p$ different patterns. This might encompass straightforward prediction algorithms such as $\hat{f}_m = E(\boldsymbol{Y}|\boldsymbol{X_M},\boldsymbol{M};\boldsymbol{\hat{\gamma}}_m)$ where $\boldsymbol{\hat{\gamma}}_m$ is the vector of estimated pattern-specific parameters or more complex algorithms such as pattern-specific random forests. While the potential exists for fitting $2^p$ different models, in practice only a small fraction of those patterns are observed. Note that $\hat{f}_m$ is fit only on the data in pattern $m$.

For comparison, denote the set of CCS models as $\{\hat{g}_1,...,\hat{g}_k \}$ for patterns $m=1,...,k$. For example, we might have $\hat{g}_m =E(\boldsymbol{Y}|\boldsymbol{X_M};\boldsymbol{\hat{\beta}_m^*})$. Note that $\hat{g}_m$ is fit using all records that have the full set of existing data for pattern $m$ and hence depends on a different parameter vector $\boldsymbol{\hat{\beta}_m^*}$. In Section 3.4, we discuss how to fit PS and CCS when a pattern is not observed or the observed data are too sparse.

The difference between $\hat{f}_m$ and $\hat{g}_m$ is illustrated in Table 2 using a linear model for continuous outcome $Y$ and two covariates $X_1$, $X_2$. There are only four missing data patterns: (i) $X_1$, $X_2$ both observed; (ii) $X_1$ missing, $X_2$ observed; (iii) $X_1$ observed, $X_2$ missing; (iv) $X_1$, $X_2$ both missing. We see in Table 2 that the estimated PS response function for $E[Y|X_1, M_1=0,M_2=1]$ is $\hat{f}_3$ while the CCS analogue $E[Y|X_1]$ is estimated by $\hat{g}_3$. Here $\gamma_{p,m}$ does not necessarily equal $\beta^*_p$ for any $m=1,2,3,4$. It is tempting to assume that the CCS pattern 1 model would yield a reliable estimate of the marginal model $E[Y|X_1, X_2]$, but this only happens when the data are MAR on the covariates (and not on the response) (see White and Carlin, 2010; Bartlett *and others*, 2014).

**Table 2.** Comparison of pattern submodels and complete case submodels

**Pattern**	**PS ($\hat{f}_m$)**	**CCS ($\hat{g}_m$)**
1:$X_1^{obs},X_2^{obs}$	$E[Y|X_1,X_2,M_1=0,M_2=0] = \gamma_{0,1} + \gamma_{1,1}X_1 + \gamma_{2,1}X_2$	$E[Y|X_1,X_2] = \beta^*_{0,1} + \beta^*_{1,1}X_1 + \beta^*_{2,1}X_2$
2:$X_1^{miss},X_2^{obs}$	$E[Y|X_2,M_1=1,M_2=0] = \gamma_{0,2} + \gamma_{2,2}X_2$	$E[Y|X_2] = \beta^*_{0,2} +\beta^*_{2,2}X_2$
3:$X_1^{obs},X_2^{miss}$	$E[Y|X_1,M_1=0,M_2=1] = \gamma_{0,3} + \gamma_{1,3}X_1$	$E[Y|X_1] = \beta^*_{0,3} + \beta^*_{1,3}X_1$
4:$X_1^{miss},X_2^{miss}$	$E[Y|M_1=1,M_2=1] = \gamma_{0,4}$	$E[Y] = \beta^*_{0,4}$

$\gamma_{p,m},\beta^*_{p,m}$ represents the effect of the $p^{th}$ covariate in pattern $m$

### 3.2. Prediction Performance of PS

PS is computationally efficient because it fits a series of models in which all the necessary data is observed. Thus, we need only fit and cross-validate the pattern-specific models using standard techniques. Moreover, minimizing the expected loss in each pattern is equivalent to minimizing the expected loss marginally.

#### 3.2.1. Minimization of the expected prediction error.

Minimizing the expected prediction error (EPE) in each pattern will, in turn, minimize the overall EPE. Let $L\left(Y,\,\hat{f}\right)$ be a properly defined loss function for outcome $Y$ and forecasting algorithm $\hat{f}$. We then have:

$$\begin{align*}E_{Y|X}[L(Y\!,\,\hat{f}(\boldsymbol{X})) ] &= E_M\left[E_{Y|\boldsymbol{X_M},\boldsymbol{M}}\left[L\left(Y\!,\,\hat{f}_m\right)\right]\right]\\
&=\sum_M P(M) E_{Y|\boldsymbol{X_M},\boldsymbol{M}}\left[ L\left(Y\!,\, \hat{f}_m(\boldsymbol{X_M},\boldsymbol{M}) \right) \right]
\end{align*}$$

where $\hat{f}_m=\hat{f}_m(\boldsymbol{X_M},\boldsymbol{M})$. Hence, the selection of $\hat{f}_m$ that minimizes the pattern specific expected loss, $ E_{Y|X_M,M}\left[L\left(Y, \hat{f}_m(\boldsymbol{X_M},\boldsymbol{M}) \right) \right]$, will in turn minimize the overall loss $ E_{Y|X}[L(Y, \hat{f}(\boldsymbol{X})) ]$.

The form of the loss function is flexible; it could be squared error loss or 0/1 loss (Hastie *and others*, 2009). The pattern-specific restriction of the loss function should not be overlooked; the result might not hold for metrics where predictions in one pattern are compared with predictions in another. One example of this is the Area under the receiver operating characteristic curve (AUC), with the overall AUC not equalling the average of pattern specific AUCs.

This result implies that, in practice, prediction models should be constructed and cross validated within each pattern in order to maximize predictive ability. Note the restriction of this result to a certain class of models $f$. This class must remain constant across patterns. If a linear model of a certain form is fit for the overall model, then a linear model of the same form must be fit for each pattern (with the obvious exception that missing predictors are excluded in the PSs). If a cross-validated relaxed lasso is used to develop the overall model, then a cross-validated relaxed lasso must be used within each pattern. Likewise for random forests. The above result does not necessarily apply when the prediction class changes across patterns or does not match the prediction class used for the overall model. Rather, the result only tells us the optimal way to deal with the missing data in that class of prediction algorithms. We explore this point using a relaxed lasso in our data example. Also, for the record, we note that while it is sometimes possible to find a more parsimonious model that yields a smaller EPE, these tend to occur in âŁ˜smallâŁ™ samples, highly collinear settings, or low signal-to-noise contexts (see Shmueli, 2010), and this is not a reason to avoid the PS approach.

In Section 1.1 in the supplementary material available at *Biostatistics* online, we revisit a simple example given by Shmueli (2010) in which the EPE is evaluated for a “fully specified model” (large) versus an “underspecified model” (small). We can visualize the EPE as a weighted average of the large and small prediction models. The EPE for the correctly specified full model is just the irreducible error, whereas the EPE for the underspecified model increases as the out-of-sample predictor moves away from its population mean.

### 3.3. Practical considerations

Forecasting with MI comes with a substantial computational burden because the imputation algorithm must be repeated with the addition of the new records to the original data. PS, on the other hand, do not need to be re-computed. The upfront computational effort can be large for $2^p$ patterns, but this is still minor relative to repeated applications of MI. A drawback of PS is that when data are sparse within a pattern, it may not be possible to fit the PS. In such cases it is necessary to make simplifying assumptions and CCS is an alternative option. This hybrid approach has worked well for us in practice, in large part because the contribution to the EPE for patterns that are sparse is often negligible.

If $p$ is very large, and storing $2^p$ prediction models is unreasonable, there are several options. First, fit models only for observable patterns, ignoring patterns not observed. Second, only fit models for patterns in which the missing variable, or combinations of missing variables, are “important” to the predictions. Third, if the data are available in real time, it may be possible to fit the specific pattern mixture submodel on demand. Lastly, an examination of the MIMI model during the model construction stage can indicate how best to borrow strength over the patterns (Section 3.4).

When the number of predictors is large relative to the number of data records, our approach is no worse than traditional approaches. There are no easy answers when the sample size is too small to permit a meaningful exploration of the predictors space. And, of course, âŁoesmallâŁž is relative to the number of missing data patterns and potential predictor space and the irreducible noise levels. In practice we use the following rule of thumb: fit the PS when there are $2p$ data points in the missing data patterns, otherwise fit a CCS. Alternatively, a lasso model might work well in these settings. When the number of predictors and the amount of data are both large (e.g. EMR data), MI can be nearly impossible to implement due to computational limitations. In these cases, the PS approach is not only feasible, it is to be preferred. It is actually easier, in a computational sense, to break the problem up into to a large number of smaller challenges that can be dealt with in parallel.

### 3.4. PS as the limit of MIMI model

The MIMI model is a MI model that is dependent on the indicators $M_i$ from $i=1,..,p$. Consider the case of a linear model with $p=2$ covariates, $X_1$ and $X_2$. In this case, we could write:

(3.1) \begin{align*}\begin{split}E[Y|X_1,X_2,M_1,M_2] = \beta_0 &+ \beta_1X_1 + \beta_2X_2 + \delta_1M_1 + \delta_2M_2 \\&+ \delta_3X_1M_1 + \delta_4X_2M_2 + \delta_5X_1M_2 + \delta_6X_2M_1\end{split} \end{align*}

where the $ \boldsymbol{\beta}$ parameters represent the traditional direct effects of interest and the $\boldsymbol{\delta}$ parameters, which we call auxiliary parameters, explain how the direct effects change according to missing data pattern. If the data are MCAR, then $\delta_i = 0$$\forall$$i$. Beyond MCAR, the traditional effects might have complex dependencies with the auxiliary parameters. An examination of the magnitude of the $\boldsymbol{\delta}$ parameters provides some insight into the apparent influence of the observed missing data mechanism. If it appears that the missing data indicators contribute little to the mean model, then the MAR mechanism may be a sufficient approximation. Shrinkage methods may also be applied to the delta $\boldsymbol{\delta}$ parameters to help assess which covariates appear to be influenced by non-ignorable missing data mechanisms. The shrinking and interplay of auxiliary parameters is the subject of ongoing work.

Molenberghs *and others*, 2008 describes a longitudinal setting where every MNAR model can be decomposed into a set of MAR models. Molenberghs *and others*, 2008 rightly asserts that this duality is problematic for inference about a parameter. However, as noted in the article, these representations yield different predictions and so the implications of this duality in our context are less problematic. The result is relevant here only in what is implied about the non-longitudinal setting: that predictions must match those from the pattern mixture model in order to retain predictive optimality.

Note that model 3.1 cannot be fit unless the missing predictors are imputed. When the missing data are imputed by a proper MI algorithm, the coefficients are identified by that algorithm’s imputation scheme and the auxiliary parameters become estimable under those assumptions. When conditional mean imputation is used for missing out-of-sample predictors in the MIMI model, direct substitution shows the subsequent forecasts are equivalent to PS predictions.

Adding missingness indicators to a model is often criticized. The classical missing-indicator approaches assigned a constant (often zero or the overall mean) for the missing values and augmented the design matrix with a binary indicator for each covariate with missing values. This leads to biased parameter estimates (Allison, 2001; Groenwold *and others*, 2012). However, when conditional mean imputation is used to impute missing predictors, the missing-indicator method will yield unbiased parameter estimates in the same scenarios where complete case estimation is unbiased (Jones, 1996; Dardanoni *and others*, 2011, 2015). That is, when $M_i$ and $Y$ are conditionally independent given $X_i$, then for any choice of imputation matrix, the ordinary least squares (OLS) estimate of (3.2) coincides with the OLS estimate of $\boldsymbol{\beta}$ in the complete case model (Bartlett *and others*, 2014; Jones, 1996; Dardanoni *and others*, 2011; White and Carlin, 2010). Thus, the MIMI model is essentially an extension of Jones (1996) to a more flexible MI setting.

The connection between the MIMI model and PS can be seen through a simple rearrangement of the mean model 3.1. PS are most easily understood as projections of the true pattern-specific model into the space of observed covariates. Applying the plug-in principle, the MIMI mean model reduces to the PS when conditional mean imputation is used to impute missing covariates. Denote the imputed covariates as $X^*_i = E[X_1|X_2] = \alpha_0 + \alpha_2X_2$ if $X_{i1}$ is missing and $X_{i1}$ otherwise.

Rearranging the MIMI model we have:

$$\begin{align*}E[Y|X_1,X_2,M_1,M_2] = &(\beta_0 + \delta_1M_1 + \delta_2M_2)\\ &(\beta_1 + \delta_3M_1 + \delta_5M_2)X_1\\ &(\beta_2 + \delta_4M_2 + \delta_6M_1)X_2\\\end{align*}$$

which is just the PS model. For illustration, we have:

$$\begin{align*}E[Y|X_1,X_2,M_1=0,M_2=0] &= \beta_0 + \beta_1X_1 + \beta_2X_2\\E[Y|X_1,X_2,M_1=1,M_2=0] &= (\beta_0 + \delta_1) + (\beta_1 + \delta_3)E[X_1|X_2] + (\beta_2 + \delta_6)X_2\\& = (\beta_0 + \delta_1)+ (\beta_1 + \delta_3)(\alpha_0 + \alpha_2X_2) + (\beta_2 + \delta_6)X_2\\& = (\beta_0 + \delta_1 + \beta_1\alpha_0 + \delta_3\alpha_0) + (\beta_2 + \delta_6 + \beta_1\alpha_2+ \delta_3\alpha_2)X_2\\& = \gamma_0 + \gamma_2X_2\end{align*}$$

Here model $E[Y|X_1,X_2,M_1=1,M_2=0] = \gamma_0 + \gamma_2X_2$ is just the submodel including only the covariate $X_2$ fit within the group of individuals who are missing the covariate $X_1$ (this is why the conditioning on $M$ is important). This is just $\hat{f}_2$ in Section 3.1. Hence, PS and MIMI are two parameterizations of the “same” mean model. There are some differences; the MIMI model forces constant variance across all missing data patterns, whereas PS as implemented here allows the variance to vary across patterns. Essentially, PS is a series of models based on the observable total effects that can be estimated from the data at hand, while MIMI tries to reparametrize those effects into pattern-specific direct effects.

## 4. Simulations

We present simulations in a simple linear model case under a wide variety of missing data mechanisms. The behavior, in principle, would extend to the generalized linear model (GLM) setting and complex prediction machines. To start, we generated $n$ multivariate normal predictor vectors according to $\big(\begin{smallmatrix} x_1 \\ x_2 \\ \end{smallmatrix}\big) \sim N(\boldsymbol{\mu},\boldsymbol{\Sigma})$, where $\boldsymbol{\mu}=(3,3)$ and $\boldsymbol{\Sigma}=\big(\begin{smallmatrix} 1 & 0.5 \\ 0.5 & 1 \\ \end{smallmatrix}\big)$, for example, are set to provide certain predictor profiles in terms of their correlation. Simulated outcomes $Y$ are generated from various combinations of $x_1$ and $x_2$. The pattern mixture model formulation uses $\boldsymbol{X}$ to induce one of three missing data mechanisms, MCAR, MAR, or MNAR. The outcome $Y$ is then generated from the MIMI mean model using the true $\boldsymbol{X}$ values and the simulated missing data indicators. Here, the missingness may only depend on the predictors vector $X$. In contrast, the selection model formulation simulates $Y$ from the marginal model $Y = \beta_0 + \beta_1X_1 + \beta_2X_2 + \epsilon$, where $\epsilon \sim N(0,1)$. Missing data indictors are then induced according to the desired mechanism. Note that here the missingness may depend on the outcome $Y$.

We simulated the following five missing data mechanisms for this situation: MCAR, MAR, MNAR, MARY, and MNARY. The latter two mechanisms could only be simulated in the selection model formulation. We forced the missingness data mechanism to be consistent between the in-sample and out-of-sample populations, and $\nu_0$ is empirically calculated to maintain the desired probability of missingness. The missing data mechanisms are described more in the Table 1 of the supplementary material available at *Biostatistics* online.

Parameters profiles explored were $\beta_1 = 1,3,5$, $\rho = 0, 0.5, 0.75$, $P(M_1 = 1) = 0.20, 0.50, 0.75$, and $n = 50, 200, 500, 1000$. We present here only one case that was largely representative of our findings: $\beta_1 = 3$, $\rho = 0.5$, $P(M_1 = 1) =0.50$, and $n = 1000$. For the out of sample population, we assumed one-by-one enrollment. Missing data was imputed by zero imputation, unconditional mean imputation, single conditional mean imputation using a Bayesian conditional mean model, single conditional mean imputation using a frequentist conditional mean model, or MI (predictive mean matching, 10 imputations). We fixed the imputation engine based on the in-sample population to closely mimic real-world application of these methods.

### 4.1. Simulation procedure

We compared the performance of PS, complete case model predictions, CCS predictions, traditional MI, and the MIMI imputation model. The full simulation procedure was as follows: (i) data are generated and missing data indicators are generated according to described; (ii) missing data are imputed; (iii) the MI model, MIMI model, CCS, and PS models are fit; (iv) step 1 is repeated to obtain a new out-of-sample population; (v) individuals are imputed one by one, using the above imputation procedures, assuming a fixed imputation engine from the in-sample population; (vi) individual predictions and performance measures are computed; (vii) steps 1 through 6 are repeated 1000 times.

A squared error loss function was used to compare performance of the approaches. For example, the squared error loss across all missing data patterns in the PS is $ \frac{1}{n}\sum_i\sum_jP(M_i =1)(Y_{ij} - \hat{Y}_{ij})^2$ where $j=1,,n$ subjects and $i=1,,m$ patterns. This loss is averaged over the 10 000 simulations to approximate the expected loss. Table 2 of the supplementary material available at *Biostatistics* online shows the average squared imputation error for predictor $x_1$ as a function of imputation strategy and missingness mechanism.

### 4.2. Simulation results

**Fig. 1.**
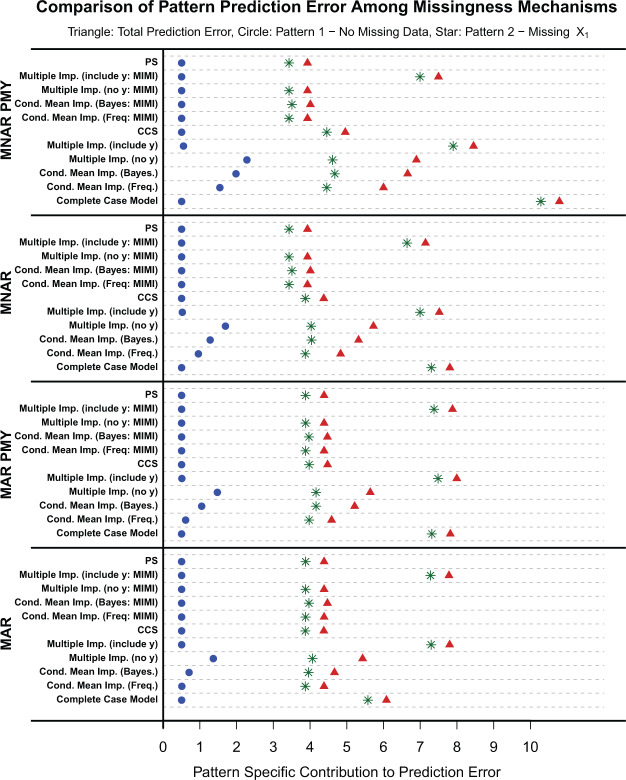
Simulation results set of parameters: $\beta_0 =1,\beta_1=3,\beta_2=1,\delta_1 = 1, \delta_3 = 1, P(M_1 =1)=0.5, \nu_1 = 1, \nu_2 = 1, \nu_{1,Y} = 1, \nu_{2,Y} = 1$. The missing data mechanisms missing at random (MAR) and missing not at random (MNAR) were generated under a pattern mixture $Y$ (PMY) and selection model $Y$ formulation. Triangles represent the total prediction error (TPE) summed over all missing data patterns. Circles represent the prediction error (PE) for pattern 1, where there is no missing data. Stars represent the PE for Pattern 2, in which x1 is missing.

Results are presented in Figure 1 for the following set of parameters: $\beta_0=1, \beta_1=3, \beta_2=1, \delta_1 = 1, \delta_3 = 1, P(M_1 =1)=0.5, \nu_1 = 1, \nu_2 = 1, \nu_{1,Y} = 1, \nu_{2,Y} = 1$. There were negligible differences in pattern-specific and total squared error loss for the MCAR and MAR missing data mechanism. Differences in the pattern-specific squared error were most apparent when $X_1$ was MNAR and MNARY (MNAR where the missingness is dependent on the outcome $Y$). For all missing data scenarios, MI and conditional mean imputations resulted in a biased parameter estimation. This bias appears most clearly in predictions for observations without missing data (blue dots). When $Y$ is added to the MI model, the model parameters had negligible bias. However, since the out-of-sample $Y$ is missing, the out-of-sample imputations of $x_1$ have greater bias than the imputation model in which $Y$ is not included resulting in a higher total prediction error (e.g. see Table 4).

When $Y$ is generated from a selection model formulation, all methods perform similarly (apart from MI as described above) under the MAR missing data mechanism. When data are MNAR, PS, and the MIMI models have slightly lower total and pattern-specific squared error loss compared with the traditionally available methods. When $Y$ is generated under the pattern mixture formulation with a MNAR missing data mechanism (MNAR PMY), PS and MIMI have both lower pattern-specific contributions to the prediction error (PE) in the pattern where $X_1$ is missing, and lower total prediction error compared with all other methods.

As might be expected, PS and CCS have different out-of-sample prediction performance when the missing data mechanism is not MAR. In fact, PS minimizes the expected prediction loss regardless of missingness mechanism, while CCS tends to rival PS only when the data are MAR. We will see that when the data are modified to induce a MNAR mechanism, PS has optimal predictions on average compared with traditional methods.

As both the strength of the missingness mechanism and the beta coefficient associated with the missing variable increase, the magnitude of the differences in methods favors PS/MIMI over all the other methods.

**Fig. 2.**
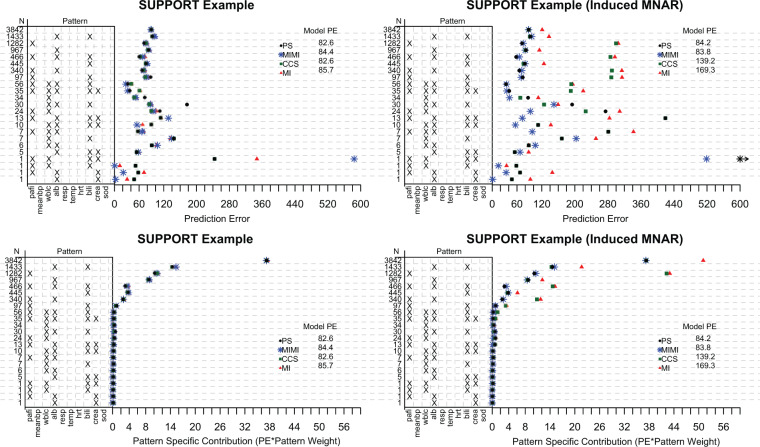
The covariates included in the SPS prediction model include partial pressure of oxygen in the arterial blood (pafi), mean blood pressure (meanbp), white blood count (wblc), albumin (alb), APACHE III respiration score (resp), temperature (temp), heart rate per minute (hrt), bilirubin (bili), creatinine (crea), and sodium (sod). There are 23 patterns present in the SUPPORT data, and missing covariates are denoted with “X”. $N$ is the total number of subjects in each missing data pattern. Pattern submodels (PS), multiple imputation with missingness indicators (MIMI), complete case submodels (CCS), and traditional multiple imputation (MI) methods are all compared. The top two figures are the unweighted pattern specific PE, and the bottom two figures are the pattern specific contribution to the PE in which the partial PE is weighted by the observed proportion of individuals in each pattern.

Table 2 of the supplementary material available at *Biostatistics* online of out-of-sample imputations of $x_1$ provides insight into some of the biases seen in Figure 2. When $Y$ is included in the imputation model during model construction, parameter estimates tend to be unbiased. When $Y$ is included during MI performed using predictive mean matching and chained equations, the imputations of $x_1$ have the largest squared error of all the imputations procedures for every missing data mechanism apart from unconditional mean imputation. Although the apparent bias in imputations for missing covariates seem small, their total contribution over all individuals can be quite significant. These results show that âŁ˜smallâŁ™ in imputing missing predictors leads to poorer downstream predictions and larger prediction error for the outcome.

Articles have explored in detail the advantages of including Y in the imputation model (Moons *and others*, 2006). Using $Y$ in the imputation model during model construction leads to unbiased estimates of regression coefficients. While this may be a fine approach during the model building stage, it is not practical in the prediction setting where the outcome is unknown. When using chained equations imputation models in which the covariate with the least amount of missing data (in these simulations $Y$) is imputed first, the next missing covariate of the chain (in these simulations $X_1$) can have very biased imputations. We do not present the situation in which $Y$ was used in the in-sample imputation model to produce unbiased regression estimates, but not included in the out-of-sample imputation model—a combination which would have less propagated imputation bias. Even though it may seem that the inclusion of $Y$ in the imputation model will lower prediction error, careful thought and attention need to be placed on the practicality of this, as well as the statistical implications.

## 5. Application: SUPPORT data example

The Study to Understand Prognoses and Preferences for Outcomes and Risks of Treatments (SUPPORT) was a multi-center, two phase study, of 9105 patients. The primary goal of the study was to model survival over a 180-day period in seriously ill hospitalized adults (Knaus *and others* (1995)). A component of the SUPPORT prognostic model was the SUPPORT day 3 Physiology Score (SPS), a risk score created to account for various sources of health variation and co-morbidities. The SUPPORT physiology score can range from 0 to 100 and was derived from the following covariates: disease group (four levels), partial pressure of oxygen in the arterial blood, mean blood pressure, white blood count, albumin, APACHE III respiration score, temperature, heart rate per minute, bilirubin, creatinine, and sodium. The SPS model allowed mean blood pressure, white blood count, albumin, temperature, HR, bilirubin, creatinine, and sodium to have a nonlinear association with SPS, and included certain interactions with disease group and albumin, and disease group and white blood count.

For our illustrative example, we choose to model SPS score because it was a known quantity. We allowed for stochastic variation by using a less sophisticated predictive model (e.g. non-linear terms and interactions were excluded and the disease group variable was dropped). This provides a controlled setting in which we can adequately assess the behavior of our predictive models. We note that obtaining a valid SPS score was important because it was the most important prognostic factor in the SUPPORT survival model.

After excluding an individual missing SPS score, and one individual missing all covariates, 9103 individuals remained of which 3842 had complete data, 2323 were missing partial pressure of oxygen in the arterial blood, 212 were missing white blood count, 3370 were missing albumin, 2599 were missing bilirubin, and 66 were missing creatinine, resulting in 23 observed missing data patterns, and 1024 possible missing data patterns. Ten-fold cross-validation was used to compare the squared error loss of MI, CCSM, MIMI, and PS within missing data patterns, as well as total average squared error loss, weighted by proportion of individuals in each pattern.

For illustrative purposes, we dichotomize SPS at the median, and compare all methods under generalized linear models with a logistic link function. We have also incorporated a relaxed lasso procedure within the PS, permitting a more adaptive fit for the forecasting algorithm. In this case, two PS may be based on the same predictor set but have different coefficients. For this set of examples, we compared logistic scoring rules and brier scores. Both the Brier score and logarithmic scoring rules are proper scoring rules used to estimate the accuracy of a risk prediction models. The Brier score is the average squared difference between the outcome and the predicted probability of risk $\mbox{BS}=\frac{1}{N}\sum_{i=1}^N(y_i - p_i)^2$, and the logarithmic score is defined as $\mbox{LS}=\frac{1}{N}\sum_{i = 1}^N\left(y_i\ln(p_i) + (1-y_i)\ln(1-p_i)\right)$, where $y_i$ and $p_i$ are the true outcome and predicted probability, respectively, for individual $i$. It is known that the Brier score does not penalize predictions that give very small probabilities when they should be giving larger probabilities, and therefore, the Brier score does not necessarily make the right decision about which method of two forecasts is better (Jewson, 2008). The logarithmic scoring rule (Log-Score) rewards more extreme predictions that are in the right direction. This score can be grossly inflated by a single prediction of probability of 0 or 1 that is in the wrong direction, and heavily penalizes classifiers that are confident about an incorrect classification. The logarithmic scoring rule is a rescaling of the gold standard optimization criteria and so in a sense it is the best accuracy score to use for binary outcomes.

### 5.1. SUPPORT example results

For each method, ten-fold cross validation of the prediction models was implemented. For the patterns with less than or equal to $N = (p + 1)*2 = 22$ subjects, the CCS was used, and the hybrid PS/CCS approach (as described in Section 3.2) was implemented. For the original SUPPORT data, all methods performed similarly both across and within patterns. In our simulation, we saw similar results when data were MAR, giving rise to the possibility that these data also follow a MAR mechanism. To exaggerate the missing data mechanism, we induced a MNARY mechanism by adding 25 units to individuals SPS scores who were missing the covariate partial pressure of oxygen in the arterial blood (pafi). This resulted in a large reduction in PE under PS compared with traditional MI methods and CCS, for the patterns in which partial pressure of oxygen in the arterial blood was missing.

The original data results are shown in the two sub-figures in the left of Figure 2. The total model PE does not differ between the four methods. When a MNAR mechanism is induced in the support data, as shown in the two left sub-figures, PS and MIMI outperform CCS and MI. In the patterns for which partial pressure of oxygen in the arterial blood (pafi) is missing, the benefits of PS and MIMI compared with CCS and MI are apparent. For these patterns, both the unweighted PE (Figure 2 top-right) and weighted PE (Figure 2 bottom-right) show this reduction in PE. The model PE, which is the sum of all the pattern specific contributions to the PE results in approximately a 50% reduction in PE for PS/MIMI compared with MI, and a 40% reduction in PE for PS/MIMI compared with CCS.

**Fig. 3.**
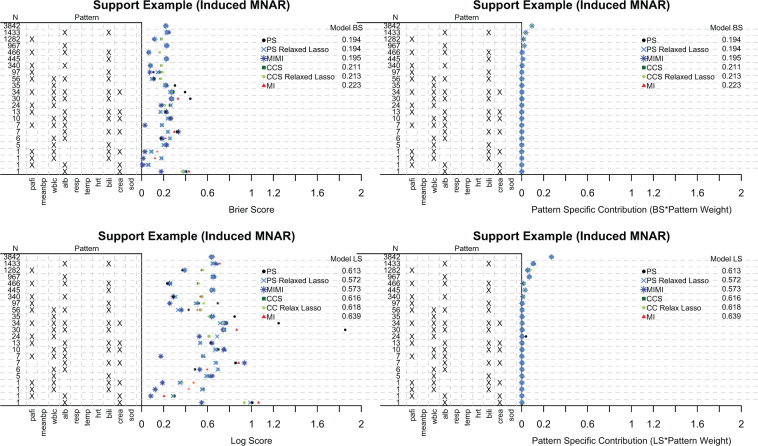
The continuous SPS measurement was dichotomized at the median, and then used as a binary outcome. The covariates included in the SPS prediction model include partial pressure of oxygen in the arterial blood (pafi), mean blood pressure (meanbp), white blood count (wblc), albumin (alb), APACHE III respiration score (resp), temperature (temp), heart rate per minute (hrt), bilirubin (bili), creatinine (crea), and sodium (sod). There are 23 patterns present in the SUPPORT data, and missing covariates are denoted with “X”. $N$ is the total number of subjects in each missing data pattern. Pattern submodels (PS), relaxed lasso PS, multiple imputation with missingness indicators (MIMI), complete case submodels (CCS), relaxed Lasso CCS, and traditional multiple imputation (MI) methods are all compared. The top two figures are the brier score (BS; unweighted pattern specific BS and weighted pattern specific BS), and the bottom two figures are the log-score (LS; unweighted pattern specific LS and weighted pattern specific LS). The prediction measures are cross-validated (10-fold), with nested cross-validation of the submodels.

The same general pattern holds when PS has an underlying logistic model. For the original SUPPORT data, both the Brier score and Log-Score are approximately equal across all methods (Figure 2 of the supplementary material available at *Biostatistics* online). In Figure 3, when a MNARY mechanism is induced, we see slightly smaller Brier scores for PS and PS fit with a relaxed lasso (PS Relaxed Lasso) compared with CCS and MI. The PS Relaxed Lasso and the MIMI model have the smallest Log-Score compared with the other methods. It seems as though the PS Relaxed Lasso potentially has an advantage over PS in the patterns with lower membership, and this ability to choose a more parsimonious model is seen in the reduction of the Log-Score since the Log-Score more heavily penalizes incorrect predictions. Note, inducing a MNARY mechanism is more challenging for a dichotomous outcome, and may not be as strong a mechanism as seen in the linear example.

## 6. Final thoughts

Statistical literature abounds with imputation methods for model inference, but there are very few practical solutions for obtaining predictions for new individuals who do not present with all of the necessary predictors. In this article, we have shown that PS provides optimal predictions for a variety of missing data mechanisms, and has large gains in computation time since external data and imputation models are no longer needed to make new predictions. PS is straightforward to implement and is easily be extended to complex prediction algorithms. In the age of big data, this is an important consideration and driving factor in most scientific contexts with big data.

As shown in our simulations and examples, there are common scenarios where the methods will perform similarly. Specifically, if there are low amounts of missing data, the missing data are from non-influential predictors, or missing data mechanisms are MCAR/MAR. In these cases, PS, MI CCS, will have very similar predictive accuracy. However, even if out-of-sample predictions are equivalent, CCS and PS both have the benefit of being computationally easy and efficient. For some specific remarks on features of PS, see Section 1.5 of the supplementary material available at *Biostatistics* online.

Care should be taken when developing clinical prediction models when missing data are present. Prediction models should be fit with PS and estimation could be conducted in the context of a MIMI model, since these methods are effectively robust to a wide variety of missing data mechanisms unlike MI and CCS methods.

## 7. Software

The SUPPORT data used in the examples, software in the form of R code and documentation is available at https://github.com/sarahmercaldo/MissingDataAndPrediction.

## Supplementary Material

kxy040_Supplementary_Materials
