# Supplementary material for: Missing data and prediction: the pattern submodel
Source: Biostatistics. 2018 Sep 6;21(2):236–52. doi: 10.1093/biostatistics/kxy040 (PMC7868046; doi:10.1093/biostatistics/kxy040)
Supplement: kxy040_Supplementary_Materials [file kxy040_supplementary_materials.pdf]

Biostatistics (2018), **0**, 0, *pp.* 1–7
doi:10.1093/biostatistics/MissingDataAndPrediction-Appendix

Supplementary Materials: Missing Data and Prediction: The Pattern Submodel

Sarah Fletcher Mercaldo*, Jeffrey D. Blume, Ph.D.

Department of Biostatistics, Vanderbilt University, Nashville, TN

sarah.fletcher@vanderbilt.edu

1. Supplementary Materials

1.1 *PS loss is a weighted average of a full and reduced model*

For a linear model the squared prediction error is a common and relevant loss function. To examine the bias-variance tradeoff in PS, it is helpful to revisit a simple example given by Shmueli (2010) in which the Expected Prediction Error (EPE) is evaluated for a "fully specified model (large) versus an "underspecified model" (small). Suppose data come from the model $f(x) = \beta_0 + \beta_1 x_1 + \beta_2 x_2 + \epsilon$ with $\epsilon \sim N(0, 1)$. When no predictors are missing we estimate the full model as $\hat{f}(x) = \hat{\beta}_0 + \hat{\beta}_1 x_1 + \hat{\beta}_2 x_2$. Here the expected prediction error (EPE) is the sum of the bias, variance, and irreducible error of the predictions or fitted values (Hastie *and others*, 2009):

$$\text{EPE}_L = E\left[\left(Y - \hat{f}(x_1, x_2)\right)^2\right] = \sigma^2 \left(1 + [1 \quad x_1 \quad x_2](X_L' X_L)[1 \quad x_1 \quad x_2]'\right)$$

where EPE_L denotes the EPE of the full model. In contrast the EPE of the underspecified or submodel is given by:

$$\text{EPE}_S = E\left[\left(Y - \hat{f}^*(x_1)\right)^2\right] = ((\gamma_0 + x_1\gamma_1) - (\beta_0 + \beta_1 x_1 + \beta_2 x_2))^2 + \sigma^2 [1 \quad x_1](X_S' X_S)[1 \quad x_1]'$$

where $\hat{f}^*(x) = \hat{\beta}^*{}_{0,1} + \hat{\beta}^*{}_{1,1} x_1$. Note that in this case $\hat{f}^*(x) = \hat{g}_2$. The EPE of the PS model

© The Author 2018. Published by Oxford University Press. All rights reserved. For permissions, please e-mail: journals.permissions@oup.com

is just a weighted average of the large and small prediction models.

$$\text{EPE}_{PS} = \sum_m P(M = m)\text{EPE}_m = \text{EPE}_L(1 - P(M)) + \text{EPE}_S P(M)$$

1.1.1 *Bias/Variance Tradeoff* We simulated the EPE in figure 1. The simulation fixes $X_1 = x_1$, and draws from the conditional distribution $X_2|X_1 = x_1 \sim N(\mu_2 + \frac{\sigma_2}{\sigma_1}\rho_{1,2}(x_1 = \mu_1), (1 - \rho_{1,2}^2)\sigma_2^2)$. The EPE for the correctly specified full model is just the irreducible error, whereas the EPE for the underspecified model increases as the out-of-sample predictor moves away from its population mean. There is bias-variance trade-off between the former approach (data are pooled across patterns but the implied prediction model is less good), and the latter (a better pattern-specific model that is estimated less precisely).

The out-of-sample prediction error from the large model is given by the green line in figure 1, and is equal to the model variance. If the data were generated from the large model and predictions were given from the small model that includes only X_2, then the expected prediction error is approximated by the purple points in figure 1.

The yellow points in figure 1 denote the prediction error that arised from the PS in this setting; $\hat{f}_1$ makes predictions when all data are available and $\hat{f}_2$ makes predictions when only X_2 is available. Clearly, PS has smaller EPE for every out-of-sample X_1. In this case the probability of missingness was 50%, $P(M_1 = 1) = 0.5$.

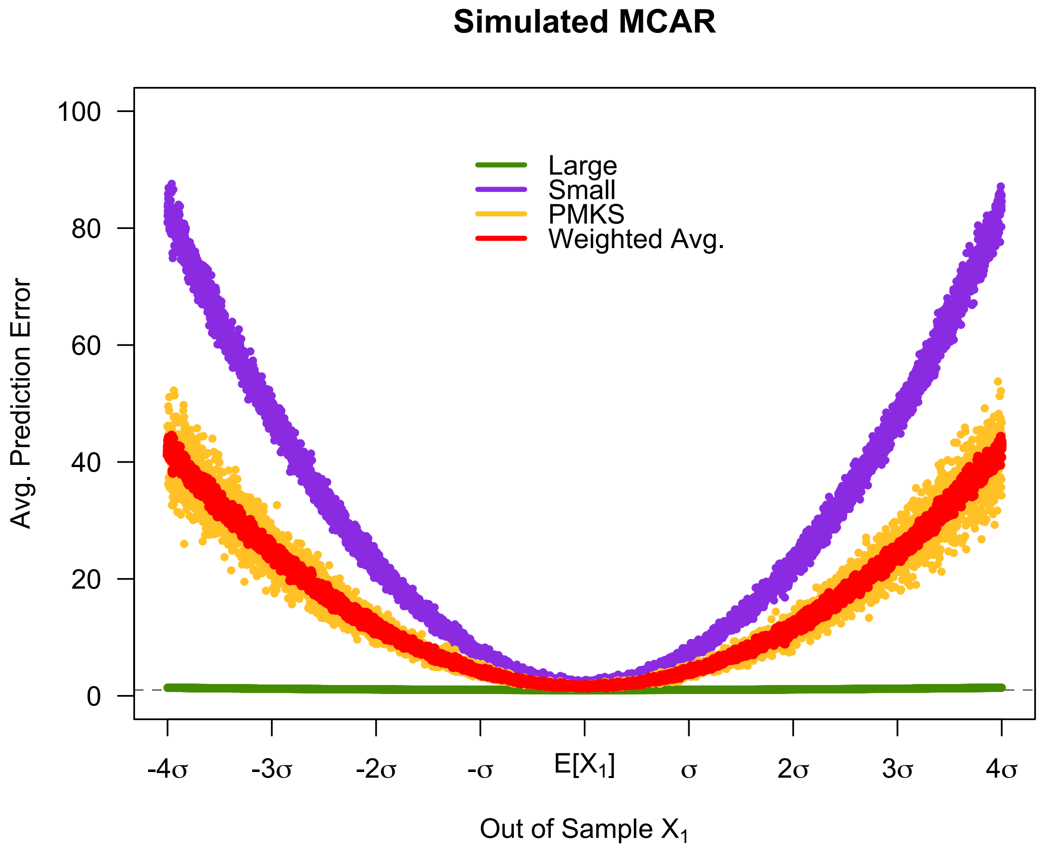

Fig. 1. Comparison of Expected Prediction Error for the Large fully specified model: $E[Y|X_1, X_2] = \beta_0 + \beta_1 X_1 + \beta_2 X_2$, and Small underspecified model: $E[Y|X_1] = \beta_{0,3} + \beta^*_{1,3} X_1$. Pattern Submodel (PS) predictions are a weighted average of the Large and Small models, weighted by P(M=1)

1.2 *Missing Data Mechanisms*

Table 1. Missing data mechanisms used for simulation. ν_0 is empirically calculated to allow the probability of missingness to maintain the desired level. $expit = \frac{e^x}{1+e^x}$.

	**Missing Data Mechanism for X_1**
**MCAR**	$P(M) = expit(\nu_0)$
**MAR**	$P(M) = expit(\nu_0 + \nu_2 X_2)$
**MARY**	$P(M) = expit\left(\nu_0 + \nu_{2,Y}\left(\frac{Y/\sigma_y + X_2}{\sqrt{2(1+cor(Y,X_2))}}\right)\right)$
**MNAR**	$P(M) = expit(\nu_0 + \nu_1 X_1)$
**MNARY**	$P(M) = expit\left(\nu_0 + \nu_{1,Y}\left(\frac{Y/\sigma_y + X_1}{\sqrt{2(1+cor(Y,X_1))}}\right)\right)$

1.3 *Imputation Error of X_1*

Table 2. Squared Imputation Error of the true out-of-sample X_1 compared to the imputed X_1 under different imputation methods and missing data mechanisms: Imputation Error of $X_1 = \sum_i (X_{1i} - \hat{X}_{1i})^2$. Multiple Impuatation was done the usual way using predictive mean matching and chained equations, where the variable with the least amount of missing data is the first variable imputed (Y), and the variable with the next least amount of missing data is imputed second (in this case X_1).

	MAR	MNAR	MAR PMY	MNAR PMY
Unconditional Mean	0.56	0.56	0.76	0.76
Cond. Mean	0.38	0.38	0.53	0.53
Cond. Mean (Bayes)	0.49	0.49	0.69	0.69
MI (no y)	0.47	0.47	0.61	0.61
MI (incude y)	0.76	0.74	0.75	0.71

1.4 *SUPPORT Example: Logistic Models*

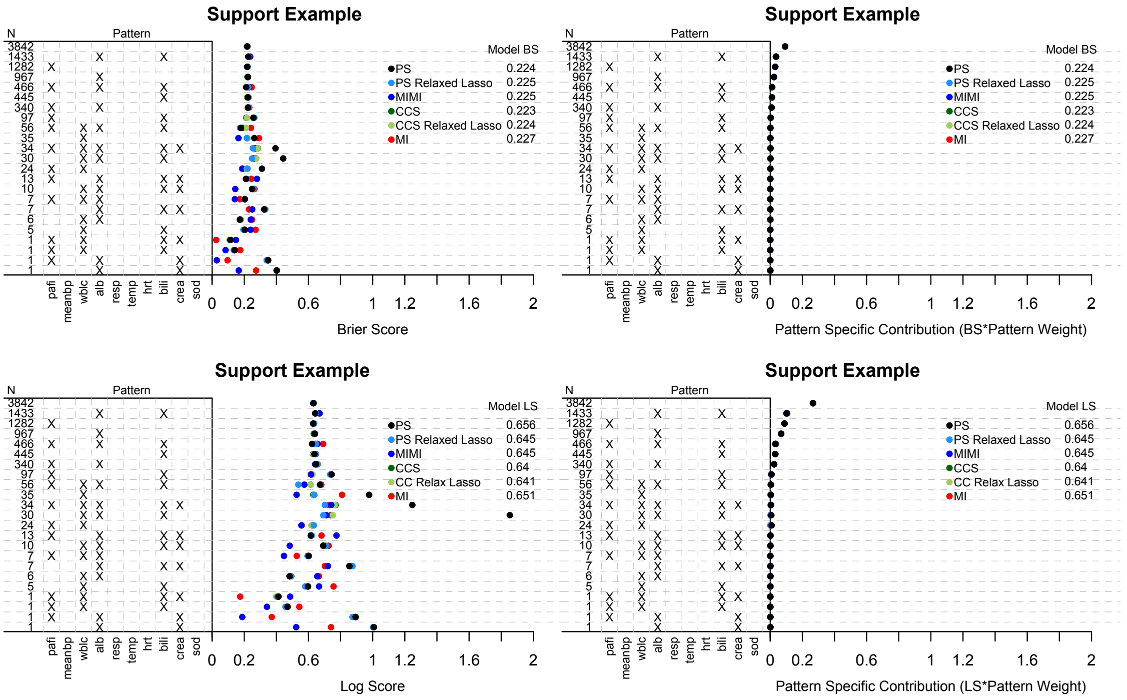

Fig. 2. The continuous SPS measurement was dichotomized at the median, and then used as a binary outcome. The covariates included in the SPS prediction model include Partial pressure of oxygen in the arterial blood (pafi), Mean blood pressure (meanbp), White blood count (wblc), Albumin (alb), APACHE III respiration score (resp), temperature (temp), Heart rate per minute (hrt), Bilirubin (bili), Creatinine (crea), and Sodium (sod). There are 23 patterns present in the SUPPORT data, and missing covariates are denoted with 'X'. N is the total number of subjects in each missing data pattern. Pattern Submodels (PS), Relaxed Lasso PS, Multiple Imputation with Missingness Indicators (MIMI), Complete Case Submodels (CCS), Relaxed Lasso CCS, and traditional Multiple Imputation (MI) methods are all compared. The top two figures are the Brier Score (BS; unweighted pattern specific BS and weighted pattern specific BS), and the bottom two figures are the Log Score (LS;,unweighted pattern specific LS and weighted pattern specific LS). The prediction measures are cross-validated (10-fold), with nested cross-validation of the submodels.

1.5 *Remarks*

1.5.1 *Remark A: Prediction vs. Estimation and Computational Efficiency*

It is important to separate goals of prediction and estimation. If prediction is the only goal, PS will always give the minimum EPE. However, if estimation is the goal, and prediction is a secondary aim, the MIMI

model can provide unbiased parameter estimates.

1.5.2 *Remark B: Conditioning Y on X and M* One might ask whether we are interested in the model marginalized over $\boldsymbol{M}$, $E[Y|\boldsymbol{X}] = \boldsymbol{X}\boldsymbol{\beta}$, or the conditional model, $E[Y|\boldsymbol{X}, \boldsymbol{M}] = \boldsymbol{X}\boldsymbol{\beta} + \boldsymbol{M}\boldsymbol{\delta}$. This is a philosophical question with differing viewpoints. For inferential purposes it has been argued that the marginal model is the model of interest. However in many situations the mixture of conditional models is the simpler way to express a complicated marginal model. Note that by first assuming the marginal model is true, one must make the assumption that data are MAR and the $\boldsymbol{\delta}$ parameters are zero. A way to assess this is to evaluate whether the degree to which the MI model and PS model give similar predictions, as we did in the SUPPORT example.

1.5.3 *Remark C: The Relationship between Y and M* The relationship between Y and M plays an important role in our modeling assumptions. An outcome generated from a selection model formulation is assumed to be independent of the missing data mechanism, such that the outcome would be the same regardless of whether covariate information is missing or observed. The pattern mixture model formulation assumes that the missing data mechanism is part of the response model, such that the outcome can be depend on the missing data pattern. The two approaches represent fundamentally different descriptions of the underlying process. And they only coincide when $\delta_1 = ... = \delta_p = 0$, which is the MAR assumption.

1.5.4 *Remark D: Extending to Generalized Linear Models and Other Prediction Approaches* We performed the same set of simulations assuming a true logistic regression model, where we used a logarithmic scoring rule to compare methods. The general ordering of results holds and will be explored in future papers. These results extend to random forests as well.

References

Hastie, Trevor, Tibshirani, Robert and Friedman, Jerome. (2009). *The Elements of Statistical Learning*, Springer Series in Statistics. New York, NY: Springer New York.

Shmueli, Galit. (2010, August). To Explain or to Predict? *Statistical Science* **25**(3), 289–310.

[*Received , 2017; revised , 2018; accepted for publication*]
